# Human mitochondrial nucleases

**DOI:** 10.1111/febs.13981

**Published:** 2017-02-01

**Authors:** Francesco Bruni, Robert N. Lightowlers, Zofia M. Chrzanowska‐Lightowlers

**Affiliations:** ^1^ The Wellcome Trust Centre for Mitochondrial ResearchThe Medical SchoolNewcastle UniversityUK

**Keywords:** cleavage, degradation, human, mtDNA, mitochondria, mtRNA, nucleases, processing

## Abstract

Mitochondria are cytosolic organelles that have many essential roles including ATP production via oxidative phosphorylation, apoptosis, iron‐sulfur cluster biogenesis, heme and steroid synthesis, calcium homeostasis, and regulation of cellular redox state. One of the unique features of these organelles is the presence of an extrachromosomal mitochondrial genome (mtDNA), together with all the machinery required to replicate and transcribe mtDNA. The accurate maintenance of mitochondrial gene expression is essential for correct organellar metabolism, and is in part dependent on the levels of mtDNA and mtRNA, which are regulated by balancing synthesis against degradation. It is clear that although a number of mitochondrial nucleases have been identified, not all those responsible for the degradation of DNA or RNA have been characterized. Recent investigations, however, have revealed the contribution that mutations in the genes coding for these enzymes has made to causing pathogenic mitochondrial diseases.

**Abbreviations**
BER: base excision repair
DNase: deoxyribonuclease
mtDNA: mitochondrial genome
PNPase: polynucleotide phosphorylase
RNase: ribonuclease

## Introduction

Mitochondria are cytosolic organelles that have many essential roles including ATP production via oxidative phosphorylation, apoptosis, iron‐sulfur cluster biogenesis, heme and steroid synthesis, calcium homeostasis, and regulation of cellular redox state. One of the unique features of these organelles is the presence of an extrachromosomal mitochondrial genome (mtDNA), together with all the machinery required to replicate and transcribe mtDNA. The accurate maintenance of mitochondrial gene expression is essential for correct organellar metabolism, and is in part dependent on the levels of mtDNA and mtRNA, which are regulated by balancing synthesis against degradation. It is clear that although a number of mitochondrial nucleases have been identified, not all those responsible for the degradation of DNA or RNA have been characterized. Recent investigations, however, have revealed the contribution that mutations in the genes coding for these enzymes has made to causing pathogenic mitochondrial diseases 1, 2.

Characterizing mitochondrial nucleases is particularly demanding as these may be present in more than one cellular compartment, for example, REXO2 is an RNase that is present in both mitochondria and cytosol. As a result, the biochemical classification of nucleases as truly mitochondrial rather than as a contaminant during purification can be challenging. Despite these difficulties, several human mitochondrial nucleases have been characterized, although the existence or the activity of some of these enzymes is still debated.

Most of the identified DNases are involved in mtDNA maintenance or repair, and almost all have both endo‐ and exonucleolytic activity, although some such as EndoG also recognize RNA as a substrate 3. The mitochondrial RNases that have been identified are, predictably, involved in mtRNA processing and degradation; however, a few may play a role in mtDNA replication but their exact function in this process is controversial. Interestingly, none of the ribonucleases characterized so far possess 5′–3′ exonuclease activity. The current catalog of mitochondrial nucleases is unlikely to be comprehensive as evidenced by recent publications, which increasingly highlight how dysfunction of these enzymes can cause pathogenic mitochondrial disease 1.

Tailoring and targeting of specific nuclease activities to mitochondria, however, is gaining increasing attention as a therapeutic tool to remove mutated mitochondrial DNA and thereby ameliorate disease 4.

**Figure 1:**
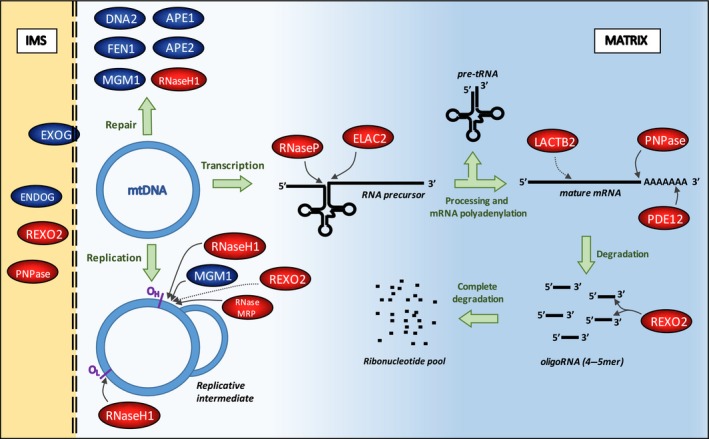
Sketch to indicate the known nucleases and their substrates in human mitochondria. The intermembrane space (IMS) is indicated in yellow and the matrix in blue. DNases are designated in dark blue and the RNases in red.

This review summarizes the recent advances in characterizing mitochondrial nucleases (Fig. 1), and the approach of engineering nucleases to be mitochondrially targeted as a tool to manipulate human mtDNA, with the long‐term goal of treating human mitochondrial dysfunction.

## Mitochondrial DNases

The number of mtDNA molecules that are found in human cells can vary from a few hundred to more than a thousand. This mtDNA is organized into complexes known as nucleoids. These structures not only include TFAM (mitochondrial transcription factor A) to package the mtDNA 5, but can also associate with other proteins and enzymes that are responsible for essential mtDNA‐related processes such as replication, recombination, and repair 6. Both the replication and maintenance of mtDNA require the activity of nucleases, mainly DNases, with different levels of specificity (Table 1). Most of the mitochondrial DNases characterized so far are involved in mtDNA repair 7 and/or the removal of primers during replication 8. Although the activity and localization of most of these enzymes has been established, whether or not they are actually involved in mtDNA repair is still debated.

**Table 1:** Mitochondrial DNases

Nuclease	Accession (UNIPROT)	Mitochondrial process(es)	Cleavage activity(ies)	Substrate		References
				DNA	RNA	
APE1	P27695	Base excision repair	Endonuclease	✓		9, 10, 11, 12
APE2	Q9UBZ4	Base excision repair	Endonuclease	✓		13, 14
			dsDNA 3′–5′ exonuclease			
DNA2	P51530	Flap intermediates processing	Endonuclease	✓		16, 17, 18, 20, 21
FEN1	P39748	Flap intermediates processing base excision repair	5′‐flap endonuclease; 5′–3′ exonuclease; RNase H activity	✓	✓	16, 19, 20, 21, 22, 23, 24
EXO G	Q9Y2C4	Single strand break repair	Endo/5′–3′ exonuclease	✓	✓	25, 26, 27
ENDO G	Q14249	Apoptosis	Endonuclease	✓	✓	28, 29, 30, 31, 32
MGME1	Q9BQP7	Flap intermediates processing base excision repair	ssDNA 5′–3′ exonuclease ssDNA 3′–5′ exonuclease	✓		33, 34, 35

One of the first DNases to be studied was AP endonuclease 1 (APE1), which is localized to both mitochondria and the nucleus 9. It has been shown to be an essential component of the mitochondrial base excision repair (BER) pathway 10, 11, which is the main repair mechanism documented in mitochondria. As an endonuclease, APE1 generates a nick at apurinic/apyrimidinic sites that are produced by DNA glycosylases during the removal of damaged bases. This cleavage leaves a 3′ terminus with a hydroxyl residue, which is used for DNA synthesis, and a 5′ terminal phosphate residue, which is subsequently dephosphorylated and ligated with newly synthesized DNA. APE1 has also been shown to have a minimal 3′ phosphatase activity 12 prompting another potential role during BER.

Another AP endonuclease shown to localize to both the mitochondria and nucleus is APE2 13. This endonuclease is thought to be involved in the BER pathway, but thus far its mitochondrial activity has not been demonstrated. APE2 also acts as a 3′–5′ exonuclease preferentially targeting mismatched 3′ nucleotides 14. This activity could potentially act during the mismatch repair to remove mitochondrial DNA fragments containing mispaired regions. Currently, this is the only enzyme that demonstrates *in vitro* 3′–5′ DNA exonuclease activity and is localized to mitochondria.

Long‐patch base excision repair (LP‐BER) occurs in human mitochondria and specifically requires a 5′‐exo/endonuclease activity 15. During LP‐BER, the strand break formation produces a 5′ single‐stranded DNA flap, which has to be processed to generate the correct DNA fragment in place for the final ligation step. In humans, the nuclease‐helicase DNA2 belongs to the PD‐(D/E)XK superfamily. It shares homology with the bacterial RecB nuclease and is localized to both the mitochondria and nucleus 16, 17. Cleavage activity assays performed *in vitro* have demonstrated a specificity for DNA intermediates that contain 5′‐flaps 18. In yeast, DNA2 interacts genetically and biochemically with FEN1 (Flap endonuclease 1), a member of the XPG/RAD2 family of endonucleases 19, and subsequent work has also confirmed their interaction in nuclei and shown that the two proteins are associated with Okazaki fragment maturation 20, 21. Interestingly, when tested *in vitro* DNA2 and FEN1 are able to increase the efficiency of flap fragment maturation 16. The interactions between these two proteins have been demonstrated experimentally in yeast, and although the involvement of FEN1 in mitochondrial BER 22 and the maintenance of mtDNA integrity has been demonstrated 23, the localization of FEN1 in human mitochondria is still debated. Recently, Holt and colleagues have also reported a shorter mitochondrial FEN1 isoform, named FENMIT, which could potentially stabilize R‐loop regions through its ability to bind, but not to cleave, RNA flap fragments 24.

It is believed that more DNases are required to complete LP‐BER. One of these, EXOG (endonuclease G‐like‐1), is implicated in the repair of single‐strand breaks in the mitochondrial genome 25. However, the depletion of EXOG in rat cardiomyocytes does not cause any loss of mtDNA integrity, although it does affect normal mitochondrial function by increasing ROS production 26. EXOG is a dimeric endo/5′–3′ exonuclease that localizes to mitochondria, specifically to the intermembrane space (IMS). The enzyme has also been found delineating the cristae, which suggests it is associated with inner mitochondrial membrane (IMM) 27. This is consistent with the distribution of mtDNA, which is packed into nucleoids that are also associated with the matrix face of the inner mitochondrial membrane.

Enzymes involved in the metabolism of mitochondrial nucleic acids are expected to localize to the matrix. Surprisingly, however, several mitochondrial nucleases have been found in the IMS and the mechanism by which these nucleases act on the mtDNA or mtRNA located in the matrix remains obscure. Endonuclease G (Endo G) is a paralogue of EXOG, which also localizes to the mitochondrial IMS 28. It belongs to the superfamily of ββα‐Me‐finger nucleases and is able to target both DNA and RNA substrates. Initially, it was postulated that EndoG was responsible for generating primers for mtDNA replication as it possesses RNase H activity 29. It has since been shown that this nuclease participates in a caspase‐independent apoptotic pathway, as the localization changes from mitochondrial to nuclear, in which compartment it promotes chromatin DNA fragmentation independently of caspases 30. Although the involvement of mitochondrial EndoG in apoptosis has been studied in different model organisms and universally accepted 30, 31, 32, its mitochondrial function still needs to be elucidated.

One of the more recently characterized human mitochondrial nucleases is MGME1, also known as Ddk1 33, 34. Like DNA2, it belongs to the PD‐(D/E)XK superfamily and shows a preferential 3′–5′ activity on single‐stranded DNA molecules. Falkenberg and colleagues have reconstituted mtDNA replication *in vitro* and using this system have demonstrated that MGME1 is able to process 5′‐flap intermediates, crucial for the RNA primer removal during mtDNA replication, into ligatable nicks in combination with DNA polymerase 35. MGME1 is clearly a constituent of the mitochondrial replisome 36, however, due to its ability to cleave 5′‐flap substrates, it is reasonable to hypothesize an additional involvement in LP‐BER during mtDNA repair process together with DNA2 and FEN1 35.

## Mitochondrial RNases

The human mitochondrial genome is transcribed as polycistronic units. This process gives rise to one short and two long transcripts. The former encompasses a ribosomal RNA transcription unit while the remaining two essentially transcribe the whole mtDNA molecule 37. Each of these transcription units needs to be processed in order to release the mt‐rRNAs, mt‐tRNAs, and the open reading frames, all of which require specific modifications to produce the mature form of the RNA species. The initial processing requires a specific series of mitochondrial ribonucleolytic activities 38 (Table 2). In recent years, different research groups have shown that when labeled by 5‐bromouridine (BrU) these RNA processing enzymes can be found together with mtRNA and other factors in well‐organized punctate structures, named RNA granules 39, 40, 41, 42. It is probable that mitochondrial RNA granules can associate, even if transiently, with transcriptionally active nucleoids to ensure an efficient coordination of synthesis, maturation, and translation of mitochondrial RNA 43, 44.

**Table 2:** Mitochondrial RNases

Nuclease	Accession (UNIPROT)	Mitochondrial process(es)	Cleavage activity	Substrate		References
				DNA	RNA	
PNPase	Q8TCS8	RNA import; mtRNA degradation	3′–5′ exonuclease		✓	45, 46, 47, 48, 49, 50, 51, 52
REXO2	Q9Y3B8	OligoRNA degradation	3′–5′ exonuclease	✓	✓	53
RNase MRP		Primer generation for mtDNA replication	Endonuclease		✓	55, 56, 57, 58
RNase H1	O60930	Primer processing mtDNA repair	Endonuclease		✓	59, 60, 62, 63, 64, 65 66
RNase P (MRPP1)	Q7L0Y3	tRNA 5′ end processing (MRPP3) tRNA methylation (MRPP1 and MRPP2)	Endonuclease		✓	77, 78 76 72, 73, 74, 75
RNase P (MRPP2)	Q99714					
RNase P (MRPP3)	O15091					
ELAC2 (RNase Z 2)	Q9BQ52	tRNA 3′ end processing	Endonuclease		✓	43, 61, 70, 71, 79, 81
PDE12	Q6L8Q7	RNA deadenylation tRNA^Tyr^ 3′ end processing	3′–5′ exonuclease		✓	38, 78, 82 83
LACTB2	Q53H82	mtRNA degradation?	Endonuclease		✓	84

One of the mitochondrial RNases that has been found in RNA granules is polynucleotide phosphorylase (PNPase) 45. The first evidence of its localization in human mitochondria was obtained more than 10 years ago when this enzyme was shown to have a cleavable mitochondrial presequence and that it differed from the cytosolic form 46. PNPase is a bifunctional enzyme, having both a 3′–5′ phosphorolytic activity and a 5′–3′ polymerase activity. Since it exhibited degradation activity, it was initially considered the ideal candidate to be the main mtRNA degrading nuclease. Since then conflicting data have been published from different laboratories generating debate about the mitochondrial function of this enzyme. While Nagaike and colleagues observed that following depletion of PNPase, there was no effect on the steady‐state levels of mt‐mRNA, only an altered polyadenylation status 47; other groups have shown that the reduced PNPase affects the level of both mature mRNAs and their precursors 48, 49. The variance in the data may result from the different approaches that were used to deplete PNPase (transient or stable RNAi interference). To further confound the understanding of its function, other studies have shown that the enzyme is localized within intermembrane space and is responsible for the import of specific cytosolic RNAs into mitochondria 49. *In vitro* assays with purified recombinant PNPase show that it forms a heteromeric complex with the helicase SUV3 and efficiently degrades structured single‐stranded RNAs 50. Further *in vitro* experiments have shown that PNPase and SUV3 can form a transient complex with the poly(A) polymerase (mtPAP) in order to modulate mt‐mRNA poly(A) tail lengths in response to energy changes 51. Finally, recent work has shed some light on the *in vivo* function of PNPase 45. This study has demonstrated that the enzyme is present and active in the mitochondrial matrix and when interacting with SUV3, constitutes the so‐called mitochondrial degradosome, which colocalizes with RNA granules.

The end product of such mitochondrial mRNA degradation is short oligoribonucleotides. PNPase is not able to degrade RNA fragments < 4 nucleotides in length 52, and if not completely degraded, these short oligonucleotides would accumulate and could interfere with either transcription or replication processes, or affect the levels of the mitochondrial ribonucleotide pool. Recently, a ribonuclease, REXO2, has been identified and characterized in human mitochondria 53. This enzyme is a member of the DEDD nuclease superfamily, named for the four conserved acidic residues that are crucial for the catalytic activity. REXO2 is a conserved exonuclease and possesses the *in vitro* ability to degrade small single‐stranded RNAs, and also DNA fragments, in a 3′–5′ direction 54. This oligo‐RNase was demonstrated to be active in human mitochondria and its depletion caused a significant decrease in both mtDNA and mtRNA levels as well as a consequential impairment of *de novo* mitochondrial protein synthesis 53. Intriguingly, REXO2 has shown a dual submitochondrial localization that is reminiscent of PNPase, as it is present in both the IMS and matrix. It is possible that this oligospecific RNase transiently interacts with mitochondrial degradosome to complement the otherwise absent degradation, however, no experimental evidence has been obtained so far. Another interesting feature is the loss of mitochondrial 7S DNA in REXO2‐depleted cells. A similar result has been found in cells overexpressing either POLγβ, the accessory subunit of mtDNA polymerase, or MGME1 34. These data, taken together with the ability of REXO2 to cleave small DNA fragments, suggests a possible role in mtDNA replication.

Almost 30 years ago, the Clayton laboratory identified and partially purified from mouse mitochondria an endonucleolytic RNase activity 55. This enzyme, named RNase mitochondrial RNA processing (RNase MRP), is a ribonucleoprotein composed of nuclear‐encoded protein and RNA elements 56. It is located in multiple cellular compartments namely, the nucleolus, cytosol, and mitochondria. Identification in these different compartments coupled with the high abundance of the enzyme in the nucleus prompted debate over the mitochondrial function. Other studies have shown that *in vitro* RNase MRP can cleave R‐loop substrates at the physiological priming sites, which would promote the mtDNA leading‐strand replication 57. In contrast, an RNase MRP‐independent mechanism for primer formation was proposed as potentially coexisting with the enzymatic cleavage model 58.

The recent hypotheses about the primer processing and removal, as part of the mtDNA replication process, invoke the formation of flap intermediates and the involvement of different mitochondrial nucleases (reviewed in 8). In addition to DNA2, FEN1, and MGME1, RNase H1 is an endoribonuclease potentially implicated in both mtDNA replication and repair. It localizes to the mitochondria showing a classic targeting sequence, but it is synthesized only from the first of the two in‐frame methionine codons in the transcript 59, 60. Intriguingly, this pattern is shared by other mitochondrial RNases that have dual or multiple cellular localizations, including REXO2 53 and ELAC2 61.

RNase H1 possesses a hybrid binding domain (HBD) that allows it to specifically digest the RNA strand of RNA/DNA heteroduplexes 62. In particular, it cleaves DNA : RNA substrates leaving a two ribonucleotide overhang at the 5′ end of the DNA, implying the need for a further nuclease activity to complete primer processing. RNase H1 has been shown to play a crucial role in regulation of mtDNA levels during embryogenesis as knockout mice show decreased mtDNA content and embryonic lethality 63. Replication of mtDNA may require long fragments of DNA : RNA hybrids as intermediates and RNase H1 activity could be indispensable for their degradation 64. Murine embryonic fibroblasts lacking RNase H1 have been shown to retain primers that are important for replication initiation both within the D‐loop and at the origin of light strand replication impeding the progression of polymerase gamma in subsequent rounds of replication 65. In addition, the importance of RNase H1 for mitochondrial genome maintenance have been recently highlighted by functional analyses performed on patient tissues and cells carrying pathological mutations in *RNASEH1* gene 66.

One of the features of mammalian mtDNA is the distribution of the tRNA genes, such that they are interspersed between the rRNA and mRNA genes. In most cases, the resulting polycistronic transcripts are processed according to the ‘tRNA punctuation model’ to release individual RNA species 67 ready for subsequent maturation. This model requires the presence of RNases with endoribonucleolytic activity that act at the 5′‐ or 3′‐end of each tRNA. Both these activities have been identified in human mitochondria and the proteins responsible, RNase P and ELAC2, respectively, have been characterized (reviewed in 68). Where there are no mt‐tRNAs between the open reading frames, there must be an mt‐tRNA‐independent mechanism in action that has currently evaded characterization (reviewed in 69), although ELAC2 involvement has been eliminated as a candidate for separating *RNA14* from *MTCO3* 70, 71.

Mitochondrial RNase P was identified as the endonuclease responsible for the removal of 5′ tRNA extensions and is composed by three protein subunits, encoded by different genes that are MRPP1, MRPP2, and MRPP3. The subunit accountable for the catalytic activity on mitochondrial tRNA 5′‐ends is MRPP3 and shows a unique feature, considering the ribozymal origin of this enzyme: in contrast with nuclear RNase P, it has no RNA component 72, 73. However, the catalytic site of MRPP3 is distorted in the absence of MRPP1 and MRPP2, its nuclease activity seems to be dependent on the other two subunits and a mouse model with a tissue specific knockout of MRPP3 has demonstrated that MRPP3 is essential 74, 75. Although MRPP2 was initially characterized as a dehydrogenase 76, together with MRPP1 it can form a subcomplex with methyltransferase activity that can modify both adenosine and guanine of most mt‐tRNAs at position 9, a modification that appears to be crucial for the correct mt‐tRNA folding 77. Further, this methyltransferase activity is preserved in fibroblasts from individuals with recessive mutations in MRPP1 gene (TRMT10C) where mtRNA processing is affected, resulting in accumulation of mtRNA precursors 78.

First identified as a candidate prostate cancer susceptibility gene 79, ELAC2 encodes an endonuclease that displays RNase Z activity, by acting on the 3′ end of tRNA molecules, but only after RNase P processing of 5′ termini 70, 75, 80. In contrast to RNase P, ELAC2 is active as a single subunit 61 and needs to be associated with mitochondrial nucleoids to initiate mtRNA processing 43. Although depletion of ELAC2 in HeLa cells caused a decreased level of several mitochondrial tRNAs, neither the mt‐mRNA/mt‐rRNA levels, mitochondrial protein synthesis nor OXPHOS were significantly affected 71. In contrast, RNA processing defects associated with mitochondrial translation and respiratory chain dysfunction have been observed in individuals carrying ELAC2 missense mutations 81. Further characterization is required to elucidate why ELAC2‐mediated endonucleolytic cleavage would require free 5′ ends.

Mitochondrial gene expression is controlled by the balance of synthesis and degradation of transcripts, which in turn modulated by other factors such as polyadenylation. Other than completion of termination codons on seven open reading frames, the function of mitochondrial poly(A) tails in mtRNA turnover and/or translation, has not been determined. The length of this modification is potentially regulated by the actions of poly(A) polymerase (mtPAP) with the LRP SLIRP complex, and the nuclease, PDE12 38, 78. This 2′‐phosphodiesterase localized to the mitochondrial matrix 82. It has both *in vitro* and *in vivo* 3′–5′ exonuclease activity, demonstrating preferential cleavage of A stretches. In addition to its function on mt‐mRNA, PDE12 has been also shown *in vitro* to trim oligo(A) tails to a single A at the 3′‐termini of mitochondrial tRNA^Tyr^, thereby completing tRNA^Tyr^, as the mtDNA sequence lacks the terminal 3′‐adenosine 83.

In this complex panorama of mitochondrial ribonucleases that act on mtRNA at different stages, neither an 5′–3′ exoRNase activity nor an endoRNase active on mitochondrial transcripts had been detected in the matrix until the report on LACTB2 (β‐lactamase‐like‐protein 2) 84. This enzyme is described as specifically targeting single‐stranded RNA substrates in *in vitro* endoribonucleolytic activity assays. In cultured cells, depletion of this protein causes an accumulation of a subset of mitochondrial transcripts and overexpression of LACTB2 has the opposite effect. However, these effects are potentially too modest for an enzyme that should be critical for mtRNA degradation in human mitochondria. Further investigations are required to confirm the *in vivo* role of LACTB2 in mtRNA processing, and to answer several remaining questions concerning missing ribonucleases including: is mt‐mRNA degradation initiated by an endonucleolytic cleavage? If so, which enzyme(s) is responsible for this cleavage? Are 5′–3′ exonucleases actually required for the complete degradation of mt‐mRNA? Since there is no tRNA gene placed between ATP8 and COX3 genes, is the maturation of ATP6/8 and COX3 mRNAs dependent upon an as yet uncharacterized nuclease?

## Targeting engineered nucleases to mitochondria: a tool to combat mtDNA‐related disorders

Since each cell contains multiple copies of the mitochondrial genome, it is possible that different mtDNA molecules, carrying mutations or polymorphisms, can coexist in the same cell. The balance between wild‐type and mutant copies, a state known as heteroplasmy, is an important factor in determining whether or not there are pathological consequences that result in mitochondrial diseases 85, 86.

**Figure 2:**
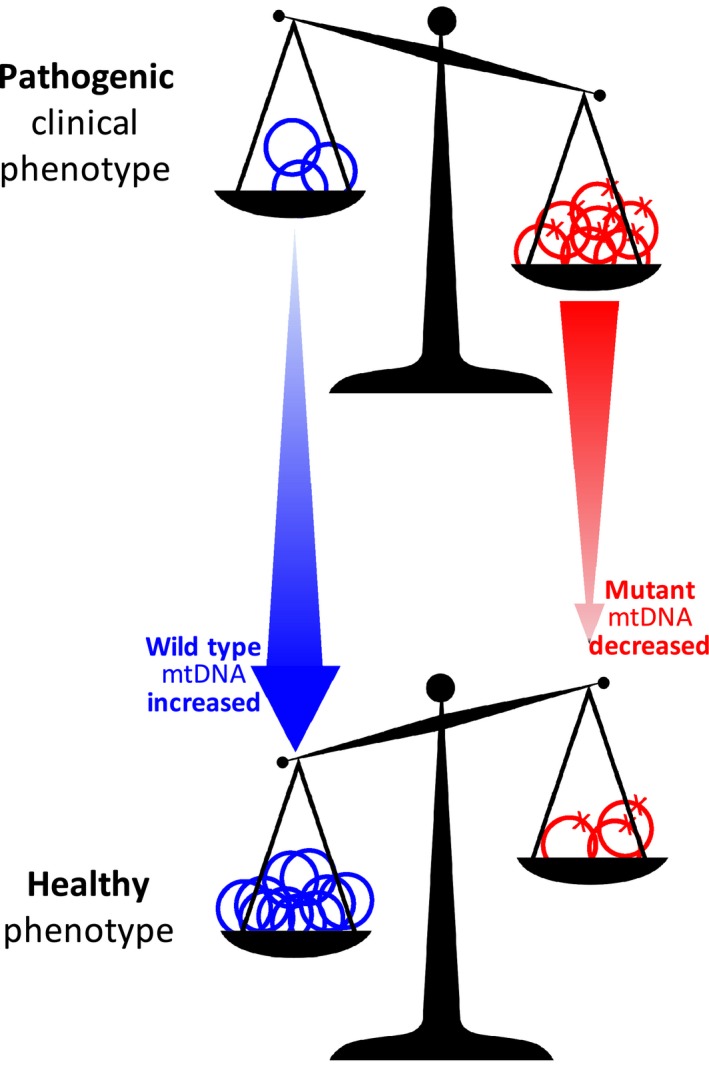
Sketch to illustrate the consequences of using different nucleases to target mutant mitochondrial DNA. Wild‐type mtDNA is depicted in blue and mutant mtDNA is depicted in red with ‘x’ indicating the presence of a mutation. The change in levels of heteroplasmy affects the clinical and biochemical phenotype.

Many approaches have been designed to try and redress the balance in favor of the wild‐type mtDNA (Fig. 2) 4. One of the most promising strategies to ameliorate mitochondrial disorders is to change the balance of mutated mtDNA molecules in favor of the wild‐type (‘heteroplasmy shift’) by expressing mitochondrially targeted endonucleases that could specifically eliminate mutated mtDNA copies and thereby decrease their number to below a ‘safe’ threshold 87. So far, three different nucleolytic activities have given promising results: restriction endonucleases (REs), transcription activator‐like effector nucleases (TALENs), and zinc finger nucleases (ZFNs). Mitochondrially targeted REs have been used for more than 15 years. These are designed to have selective recognition of restriction sites that have been created as a consequence of the mutation within the mitochondrial genome. TALENs and ZFNs are both designer nucleases consisting of a specific modular DNA‐binding domain (TALE and zinc finger, respectively) and a sequence‐independent nuclease domain. The nuclease activity is provided by the restriction enzyme FokI, which needs to dimerize to cleave DNA; however, the FokI domains are generally modified to prevent off‐target effects due to self‐dimerization. This allows the specific targeting of mtDNA sequences by engineering TALE and zinc finger domains that are different in the modularity of DNA recognition 88.

To date, mitochondrial REs have been successfully expressed in several cellular systems, and in mice, by use of adenoviral transduction. Constructs that have been injected in specific mouse tissues in this manner have shown convincing switches in the levels of heteroplasmy 88. RNA ‘restriction endonucleases’ have also been engineered and targeted to mitochondria with the different aim of silencing mitochondrial gene expression at the transcript level 89. A limiting factor in the use of restriction nucleases to decrease the mutant load of mtDNA is that mutations have to introduce specific sequences that are recognized by the enzymes as restriction sites. To circumvent this problem, a range of mitochondrial nucleases have been successfully designed and validated 4, 90, 91, 92, 93, thus exhibiting the potential to target any mutated mtDNA sequence.

All the experimental works using mitochondria‐targeted endonucleases have been performed *ex vivo* on cultured cells or *in vivo* on mice. The expression of these nucleases in patients' tissues, together with emergent genome editing technologies, has a strong therapeutic potential and may constitute a powerful tool to improve the health of people affected by mitochondrial disease.

## Conflicts of interest

The authors have no conflicts of interest to be disclosed.

## Author contributions

FB, RNL and ZCL were all involved in writing the review.
